# Compressed Air in the Treatment of Ulcers and Fistulas1Read before the Fifteenth Annual Meeting of the Iowa State Veterinary Medical Association, Cedar Rapids, Iowa, January 14 and 15, 1903.

**Published:** 1903-03

**Authors:** Arnott A. Adamson

**Affiliations:** Newton, Iowa


### COMPRESSED AIR IN THE TREATMENT OF ULCERS AND FISTULAS.[1]

By Arnott A. Adamson, M.D.V.,
NEWTON, IOWA.

Compressed air has for some time been used with varied success by physicians of this and other countries, not for its therapeutic value alone, but as a means for the application of the therapeutic agents indicated in the treatment, and for application to delicate and remote tissues that could not be reached by the ordinary methods.

In the arena of the veterinarian the successes of the physicians along these lines can be duplicated, and more. As a means of application of the required therapeutic agents in hospital practice it is convenient and exceedingly valuable in the treatment of fistulas and sinuses of a tortuous nature that will not permit the use of the knife or probe. In the application of the required pressure the medicinal agents used for the cleansing and dressing can be forced to the utmost ramification of the sinus, thoroughly cleansing it without injury to the surrounding tissue.

In the treatment of diseases of the nasal passages and respiratory apparatus it is far superior to fumigation as a means of reaching and relieving the delicate tissues and membranes in those parts that are so sensitive to anything foreign. Another advantage is that the exact amount of dosage is known, and the results arrived at in a much shorter time. Minute ulcers, almost microscopic, on the eyelids can be sprayed thoroughly and effectively, and, to a great

[1] Read before the Fifteenth Annual Meeting of the Iowa State Veterinary Medical Association, Cedar Rapids, Iowa, January 14 and 15, 1903.

advantage, with a spray. So gently is it accomplished that the patient is not discomforted in the least, as the air, when producing a spray, diffuses the force, while in the syringe or dropper the force is concentrated, it matters not how gently we endeavor to use it.

From an economical standpoint it permits the use of more of the refined and valuable instead of the cheaper and lesser refined drugs and drug preparations, reducing the waste to an absolute minimum.

